# Heritable Genome Editing with CRISPR/Cas9 in the Silkworm, *Bombyx mori*

**DOI:** 10.1371/journal.pone.0101210

**Published:** 2014-07-11

**Authors:** Wei Wei, Huhu Xin, Bhaskar Roy, Junbiao Dai, Yungen Miao, Guanjun Gao

**Affiliations:** 1 School of Life Sciences, Tsinghua University, Beijing, China; 2 College of Animal Sciences, Zhejiang University, Hangzhou, China; National University of Singapore, Singapore

## Abstract

We report the establishment of an efficient and heritable gene mutagenesis method in the silkworm *Bombyx mori* using modified type II clustered regularly interspaced short palindromic repeats (CRISPR) with an associated protein (Cas9) system. Using four loci *Bm-ok*, *BmKMO*, *BmTH*, and *Bmtan* as candidates, we proved that genome alterations at specific sites could be induced by direct microinjection of specific guide RNA and Cas9-mRNA into silkworm embryos. Mutation frequencies of 16.7–35.0% were observed in the injected generation, and DNA fragments deletions were also noted. *Bm-ok* mosaic mutants were used to test for mutant heritability due to the easily determined translucent epidermal phenotype of *Bm-ok-*disrupted cells. Two crossing strategies were used. In the first, injected *Bm-ok* moths were crossed with wild-type moths, and a 28.6% frequency of germline mutation transmission was observed. In the second strategy, two *Bm-ok* mosaic mutant moths were crossed with each other, and 93.6% of the offsprings appeared mutations in both alleles of *Bm-ok* gene (compound heterozygous). In summary, the CRISPR/Cas9 system can act as a highly specific and heritable gene-editing tool in *Bombyx mori*.

## Introduction

The silkworm *Bombyx mori* is a model organism for lepidopteran research and an important economic species due to the high demand for silk products. Despite its importance, genomic research lags behind other models such as *Drosophila*, zebrafish, yeast, and *Arabidopsis*, and a complete genetic variation map was not constructed until 2009 [1]. The biological functions of most of the genes remain elusive, particularly those related to silk protein synthesis, organ development, and cellular immunity. Although limited gene targeting with baculovirus is available in silkworm, there is an urgent need to develop further genetic engineering methods to improve research on gene function [2].

Zinc-finger nucleases (ZFNs) and transcription activator-like effector nucleases (TALENs) have been used for genomic manipulation in *Bombyx mori* [3], [4]. However, ZNFs and TALENs are based on engineered nucleases, which need time-consuming subclone and preselection processes [5]. *PiggyBac* transposon-derived vector may have a random insertion problem, and RNAi sometimes can not completely disrupt the gene's function [6]. CRISPR/Cas9 system was recently used for successful genome editing in a wide range of organisms including yeast [7], nematodes [8], [9], fruit flies [10]–[12], zebrafish [13], mammalian cell lines [14]–[16], mice [17], and plants [18]. It reveals that the CRISPR/Cas9 system is a promising strategy for genetic modification in *Bombyx mori* [6], [19]. Cas9 endonuclease is directed to the desired genomic site by a 20 bp single-strand guide RNA (sgRNA), and produces double-strand breaks (DSBs) at the target site. This activates non-homologous end joining (NHEJ) pathway in cells, which introduces random mutations at the cleavage site [20].

The aim of this study was to develop the CRISPR/Cas9 system as a highly specific and efficient technique to induce heritable gene alterations in *Bombyx mori*. We chose four silkworm genes as candidates to test our method as follows: 1) The *Bm-ok* gene (AB780443), which prompts a change in the larval cortex from translucent to turbid and also prompts the later development of an oily skin phenotype. This phenotype is easily determined visually, making it an ideal gene-targeting test locus [21], [22]. 2) The *Kynurenine 3-monooxygenase* (*BmKMO*) gene (AB063490), whose protein has a role in larval malpighian tubules and pupal ovary formation [23]. 3) The *Tyrosine hydroxylase* (*BmTH*) gene (AB439286), which can also affect insect body color and the innate immune response [24], [25]. 4) The *Bmtan* gene (AB499125). This encodes the Tan protein, which is involved in silkworm pigment formation [26], [27]. These four genes are of significant economic importance due to their associations with development, fertility, and immunity in *Bombyx mori*.

In this study, we carefully designed sgRNAs for each gene and microinjected embryos with a mixture of synthetic Cas9-mRNA and the appropriate sgRNA. Our experiments proved the effectiveness of the CRISPR/Cas9 system in *Bombyx mori*, which will greatly facilitate the functional dissection of the *Bombyx mori* genome in the near future.

## Methods

### Silkworm strains

The P50 silkworm strain was used in our experiment. It was treated for preventing diapause according to Zhao *et al*. (2012) method [28]. This strain was maintained at Zhejiang University (Hangzhou, China). Larvae were reared on fresh mulberry leaves under standard conditions at 25°C in rearing tray.

### Target design and *in vitro* synthesis of sgRNAs and Cas9-coding mRNA

Eight DNA fragments within the exons of the four genes were chosen, with 5′-GGG/A-N17/18-NGG-3′ sequences as targeting sites, according to the sgRNA recognition guidelines described by Yu *et al*. (2013) for targeted genome mutagenesis in *Drosophila* [12]. The customized sgRNAs were prepared as previously described [14], [29] (Figure S1C) with transcription performed using a RiboMAX Large Scale RNA Production Systems-T7 Kit (Promega, USA). The target sites and sequences of designed sgRNAs are listed in Table S1. Cas9-mRNA was synthesized *in vitro* using a mMESSAGE mMACHINE SP6 Kit (Ambion, USA) according to previous instructions [12]. Poly(A) signals were added to the 3′ end of capped mRNAs using *E. coli* Poly(A) polymerase Kit (New England BioLabs, USA) following the manufacturer's protocol.

### Microinjection and Cas9/sgRNA-mediated mutation screens and analysis

Fertilized eggs were collected within 1 hour of oviposition and microinjection was performed within 4 hours. As showed in Table S1, except *BmKMO* had only one target sgRNA, other 3 genes were targeted by mixed sgRNAs (2 for *Bm-ok* and *Bmtan*, 3 for *BmTH*) simultaneously. The Cas9-coding mRNA and total sgRNAs were mixed at final concentrations of 500 ng/µl and 250 ng/µl (about 2–3nl/egg), respectively. Silkworm eggs were microinjected according to the standard protocol [30] and the injected eggs were incubated at 25°C for 9–10 days until hatching.

To calculate the efficiency of Cas9/sgRNA-mediated gene alteration in the injected generation (G_0_), we collected individual specimens 8 days after injection to extract enough genomic DNA for PCR and sequencing. The four primer pairs used are shown in Table S2. Multiple sequencing peaks at a base position were indicative of mutation [12]. When multiple peaks were observed, mixed-individual PCR samples were TA-cloned and sequenced to determine the exact mutation type. In addition, for the *Bm-ok* locus, the change of skin color was used as a marker for mutation in both the injected silkworms (G_0_) and the crossed offsprings (G_1_).

### Analysis of germline mutation frequency in *Bm-ok* locus

Crossing was used to test whether mutations in injected G_0_ could be germline transmitted to the G_1_ offspring, and also to calculate the probability of heredity. Two crossing strategies were used. First, injected *Bm-ok* G_0_ moths were crossed with un-injected wild-type moths to determine the frequency of germline transmission in G_1_ (Crossing Strategy 1; Figure S2A). Second, mosaic individuals were crossed with each other to acquire G_1_ offsprings whose both alleles were mutated but at different locations quickly (compound heterozygotes. Crossing Strategy 2; Figure S2B).

### Potential off-targeting detection

The Off-targeting effect of the CRISPR/Cas9 system in silkworm was evaluated like this: First, all the potential off-target sites of the two *Bm-ok* target loci against the whole silkworm genome were searched using CasOT searching tool [31]. Second, PCR amplification to the corresponding off-target sites and DNA sequencing were performed for the evaluation of the off-targeting effects.

## Results

### The CRISPR/Cas9 system introduces mutations in silkworm with high efficiency

**Figure 1:**
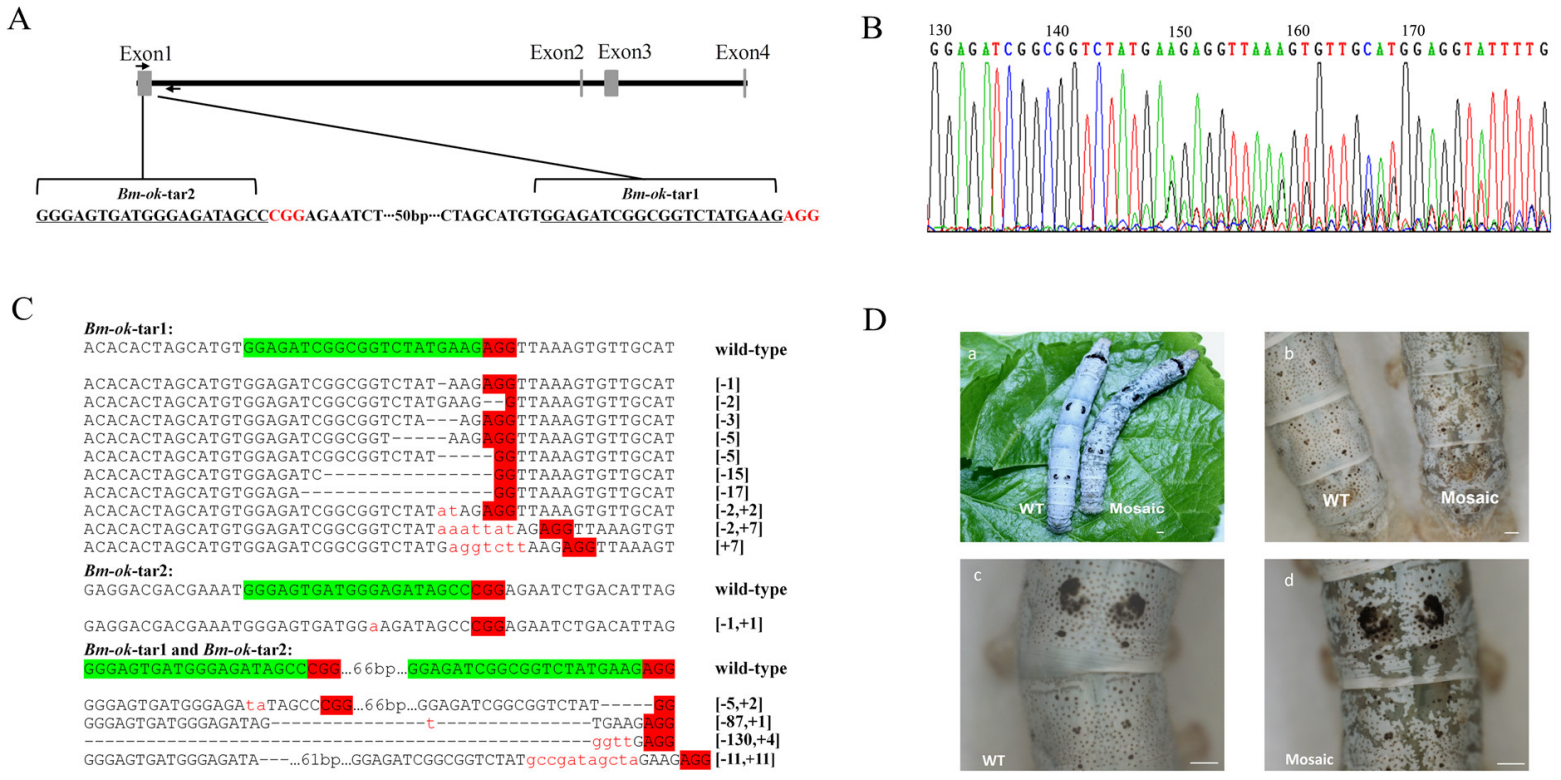
Cas9/sgRNA-induced mutations at the *Bm-ok* locus in G_0_ *Bombyx mori*. (A) Schematic representation of *Bm-ok*. exons are shown as boxes and arrows represent the primers used to amplify the target regions. Target site locations (*Bm-ok*-tar1 and *Bm-ok*-tar2) are underlined and PAM sequences are shown in red. (B) Representative chromatograms of PCR-product sequencing in G_0_ silkworms in which indel mutations are present. (C) Sequences of indel mutations at the targeted *Bm-ok* locus in G_0_ silkworms. The target sites are highlighted in green and PAM sequences are shown in red. Deletions are indicated by hyphens and insertions are shown in lowercase red letters. The indel mutation type is noted to the right (+, insertion; -, deletion). (D) Images indicating characteristic *Bm-ok* mosaic mutation phenotypes: (a) Fifth instar G_0_ wild-type (P50) (left) and mosaic mutant (right) individuals; (b–d) Magnified images of wild-type and mutant individuals. The scale bars represent 1 mm.

We selected *Bm-ok* as a candidate gene to test whether the CRISPR/Cas9 mutagenesis system could work in silkworm because of the striking skin phenotype alteration that arises upon disruption of this gene. We injected a mixture of Cas9-coding mRNA and sgRNAs targeted against two sites within exon 1 into silkworm eggs. Genomic DNA, followed by PCR and sequencing, showed that 6 of the 19 examined larvae had alterations at the target sites. Moreover, 6 of the 25 hatched larvae showed a mosaic skin phenotype 3 days after 1^st^ instar. The overall mutagenesis frequency was 31.6% in the injected generation (G_0_) (Figure 1 and Table 1).

**Table 1:** Mutations induced by Cas9/sgRNA microinjection in G_0_.

Target locus	Phenotype in examined G_0_		Frequency of mosaic in G_0_ (*n/n*)	Genotype in sequenced G_0_		Frequency of mutation in G_0_ (*n/n*)
	Mosaic (*n*)	Normal (*n*)		Mutants (*n*)	Wild-type (*n*)	
*Bm-ok*	6	19	24% (6/25)	6	13	31.6% (6/19)

Following the successful disruption of *Bm-ok*, we designed appropriate sgRNAs for *BmKMO*, *BmTH*, *Bmtan* and injected eggs as previously described. Then the mutation frequencies were analysed by sequencing of larvae bearing mutation in G_0_ stage. As showed in Table S3, the frequency of mutagenesis in G_0_ was 35.0%, 33.3%, and 16.7% for *BmKMO*, *BmTH*, and *Bmtan*, respectively (Figure S3, S4 and S5).

### The CRISPR/Cas9 system induces a variety of mutation types at target loci

Target genes were PCR-amplified from genomic DNA of larvae. PCR products were then TA-cloned and sequenced to determine the specific mutations induced by the CRISPR/Cas9 system, presumably as a consequence of NHEJ activated by Cas9/sgRNA-mediated DSBs. Usually point mutations, small insertions and deletions were the most common types of mutation in all four genes, but large fragment deletions were also found occasionally. For the *BmKMO* gene, we only focused on one site in exon 4, but deletions as long as 240 bp and 289 bp were found at the target location (Figure S3). With two target sites located in exon 1, the largest *Bm-ok* gene interruption was 130 bp (Figure 1). Three target sites were used for the *BmTH* gene, and large fragment deletion was concomitantly more frequent: a 646-bp deletion occurred between target site 1 (*BmTH*-tar1) and target site 3 (*BmTH*-tar3) that was accompanied by a 20-bp random insertion in the middle target site 2 (*BmTH*-tar2), and a 627-bp deletion occurred between *BmTH*-tar1 and *BmTH*-tar3 that was accompanied by a 35-bp insertion in *BmTH*-tar2. These data indicate that NHEJ induced by DSBs repairs DNA in such a manner as to produce a number of different types of mutation at the target sites (Figure S4).

Usually, one double strand break orientated by a proper sgRNA can induce small DNA fragment deletion or insertion in target site by NHEJ pathway. We propose that two sgRNAs can make larger DNA fragment deletion. In fact, in this study, an 87-bp fragment knockout was found in *Bm-ok* exon 1, between target site 1 (*Bm-ok*-tar1) and target site 2 (*Bm-ok*-tar2). Meanwhile, fragment knockouts as large as 281 bp occurred in *BmTH* mutant individuals, between *BmTH*-tar2 and *BmTH*-tar3. These data suggested that Cas9-induced gene locus knockout occurred.

### Cas9-induced mutations are heritable

**Figure 2:**
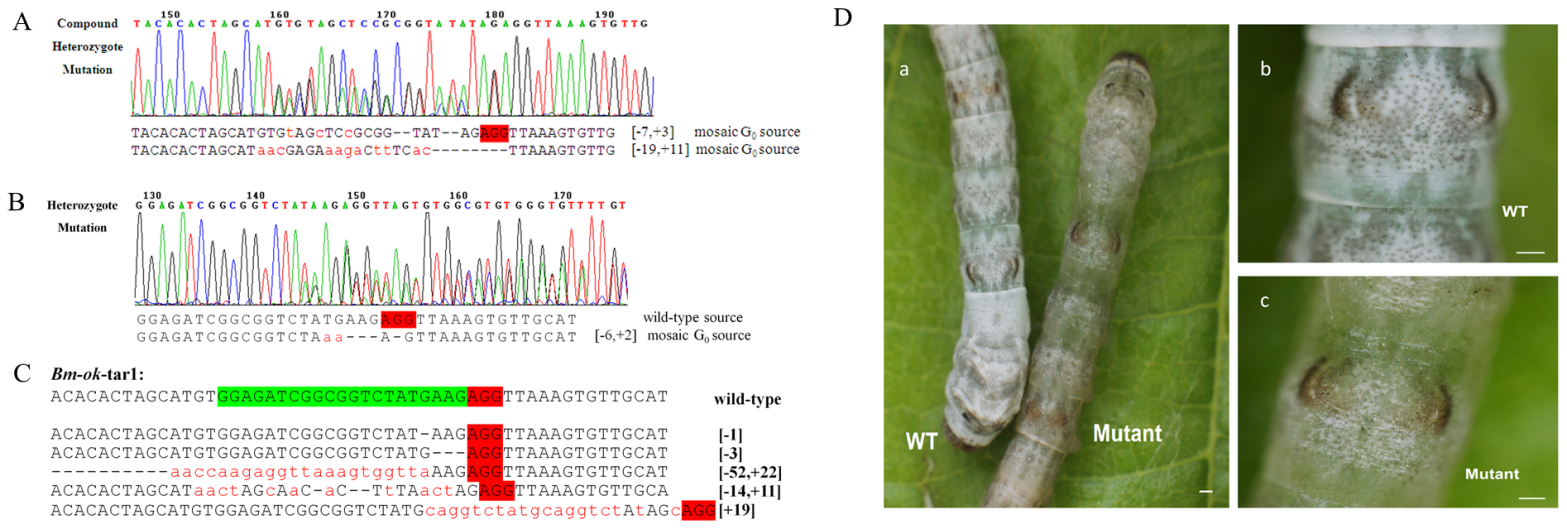
Germline transmission of the *Bm-ok* gene mutation in G_1_ progeny. Representative chromatograms of G_1_ compound heterozygous (A) and heterozygous (B) individuals generated by Crossing Strategy 2. Two peaks are apparent at several sites. (C) Sequences of individual TA-clones indicating mutations at the first target site of *Bm-ok* (*Bm-ok*-tar1) in G_1_ progeny. (D) Characteristic images indicating *Bm-ok* mutant individuals (the first day of 3^rd^ instar). (a) G_1_ wild-type (left) and mutant (right) individuals; (b) Magnified image of wild-type individual; and (c) Magnified image of mutant individual. The scale bars represent 1 mm.

We used *Bm-ok* mosaic silkworms to test for germline transmission of mutations because of their clearly determined skin phenotype. In Crossing Strategy 1 (Figure S2A) *Bm-ok* mosaic moths were crossed with wild-type moths. In this cross, 366 eggs were produced and 62 G_1_ larvae hatched. Sequencing of 28 randomly selected individuals revealed 8 heterozygotes and 20 wild-type silkworms, indicating that NHEJ-induced mutations can be transmitted to the G_1_ generation (Figure 2B and 2C). In the case of *Bm-ok*, the germline transmission frequency was 28.6% (Table 2).

**Table 2:** G_1_ germline mutations from Crossing Strategy 1.

Crossing Strategy 1	Genotype in sequenced G_1_			Frequency of germline transmission (*n/n*)
	Homozygote (*n*)	Heterozygote (*n*)	Wild-type (*n*)	
G_0_ × Wild-type	0	8	20	28.6% (8/28)

### Compound heterozygous mutants are developed from Cas9-induced mosaics by one-step breeding

To quickly obtain two alleles mutations of the same gene, in Crossing Strategy 2, *Bm-ok* mosaic G_0_ moths were crossed with siblings (Figure S2B). In total, 44 larvae with translucent epidermal phenotypes were observed from 47 offsprings, indicating a probability of homozygote production of 93.6% from crosses between mosaic silkworms of pronounced phenotype (Figure 2A and 2D; Table 3). Both alleles of *Bm-ok* gene were mutated, but the sequences might be different, we used this line as compound heterozygous mutant.

**Table 3:** G_1_ germline mutations from Crossing Strategy 2.

Crossing Strategy 2	Phenotype in examined G_1_		Frequency of compound heterozygote in G_1_ (*n/n*)
	Translucent (*n*)	Normal (*n*)	
Mosaic × Mosaic	44	3	93.6% (44/47)

### Off-targeting effects are evaluated in the injected G_0_ silkworms

Off-targeting is a potential issue about the application of Cas9/sgRNA technology in genome editing. It has been reported that PAM type and mismatch site from the seed region or non-seed region have an influence on the specificity of the CRISPR/Cas9 system [14], [32]. According to the instructions of CasOT tool designed by Xiao *et al*. (2014), we selected the top four most similar sites against the two target sites of *Bm-ok* gene for off-targeting evaluation. By using the genome template of injected G_0_ silkworms, PCR amplification to the corresponding potential off-targeting sites and DNA sequencing were performed for the detection of the off-targeting effects. Multi-peaks in sequence results are an indicator for non-specificity cleavage effects of Cas9 endonuclease [12]. In our test, we did not observe any obvious off-targeting effects (Figure S6), indicating that the application of the CRISPR/Cas9 technique could have high specificity.

## Discussion

### Differences between injected G_0_ larvae and G_1_ larvae

In this study, direct microinjection of a mixture of Cas9-mRNA and sgRNA into silkworm eggs successfully induced targeted mutations, indicating that the CRISPR/Cas9 system can act as a powerful genome-editing tool in *Bombyx mori*. In the G_0_ stage, somatic and/or germ cells were mutated randomly and not all cells were affected. Thus, *Bm-ok*-injected G_0_ animals exhibited a mosaic appearance due to a mixture of translucent mutant cells and normal non-mutant cells. To develop stable altered strains, the mutated chromosome must be present in a germline cell and then be passed to the next generation. Upon germline transmission of traits with an easily determined phenotype, such as the *Bm-ok* mutation, only homozygotes display an obvious mutant phenotype because the wild-type and heterozygous silkworms still have at least one non-mutant allele to implement normal function. This allows stable mutant homozygotes to be easily selected.

### Rapid breeding of compound heterozygotes by Crossing Strategy 2

Successful germline transmission of desired traits from mosaic mutants is critical for the development of stable lines for ongoing research. After inducing mutation with sgRNA/Cas9, we were able to efficiently generate G_1_ compound heterozygous strains by directly crossing mosaic G_0_ silkworms (Crossing Strategy 2). The editing capacity of the CRISPR/Cas9 system, combined with this crossing strategy, is a powerful and efficient method for acquiring large numbers of compound heterozygous mutant individuals. The successful implementation of this strategy requires a sufficient mutagenesis frequency and/or an easy way to identify the mutant individuals after microinjection. To test our mutagenesis and crossing strategies, we used *Bm-ok*, which produces translucent skin when mutated. In Crossing Strategy 2, mosaic G_0_ mutants (with normal and translucent skin patches) were crossed. A total of 47 G_1_ silkworm larvae were produced, of which 44 showed a completely translucent skin phenotype indicative of homozygosity. Therefore, we were able to obtain compound heterozygous mutants in a one-step cross.

### Both alleles mutated offsprings could be generated through alternative crossing strategy

While this article was under construction, Wang *et al*. (2013) reported their successful use of CRISPR/Cas9 to induce germline mutations in the *BmBLOS2* gene of *Bombyx mori* [19]. However, that study used an alternative strategy to generate homozygous mutations. G_0_ mutated mosaic individuals were crossed with wild-type moths, and mutant offsprings were determined by skin translucency (this resembles our Crossing Strategy 1). Wang *et al*. (2013) subsequently crossed G_1_ mutants to produce G_2_ homozygotes; this strategy is thus a two-step process that contrasts to our one-step process. In addition, the homozygotes produced in G_2_ of Wang's study differed from those G_1_ offsprings produced in our Crossing Strategy 2. For our compound heterozygotes, distinct mutant genes were inherited from both mosaic parents, while in the crossing strategy of Wang *et al*. (2013), the mutant locus had a single source, namely, the mutant parent in the G_1_ cross.

### Factors that influence gene targeting using the CRISPR/Cas9 system

The CRISPR/Cas9 system is a convenient manipulative method for introducing genomic changes at high efficiency and with minimal species limitation. Successful application of the CRISPR/Cas9 system requires the consideration of several factors. First, the targeting site must be carefully chosen. Different targeting sites may influence the cleavage efficiency of Cas9 protein because spatial structure disparity in chromosomes may play a role in sgRNA recognition. Second, the *in vitro* synthesis of sgRNA and mRNA for Cas9 must be performed in RNase-free environment. Third, silkworm microinjection is a skilled technique in which injection position, injection depth, and needle size can have a crucial impact on the survival rate and variation in G_0_. Although we tested our method using a gene with an easily determined phenotype, mutations in many loci do not have such visual phenotypic effects, making rapid screening more challenging. Thus, it is clear that a customized CRISPR-Cas9 system can be used to efficiently induce targeted mutagenesis in *Bombyx mori*, but it is also clear that the method requires optimization for the rapid screening of loci that are more difficult to screen.

Taken together, our data indicate that the CRISPR/Cas9 system can be an efficient tool for genome engineering technology in *Bombyx mori*. Design of sgRNA, microinjection of a customized sgRNA/Cas9-mRNA mixture, and an effective crossing strategy are the three key components for successful acquisition of a homozygous mutant. In addition, if at least two target sites were designed against a gene, it was possible to knockout gene fragments in a specific manner. The efficient gene targeting and crossing techniques developed in this study will be valuable for future silkworm genome research.

## Supporting Information

Figure S1 **Schematic illustration of the CRISPR/Cas9 system and the genome engineering process.** (A) Map of the pSP6-2sNLS-SpCas9 vector. (B) Map of the pMD19-T sgRNA scaffold vector used to produce sgRNA. The transcription of Cas9 flanked by two nuclear localization sequences (NLS) is driven by the SP6 promoter. sgRNA transcription is driven by the T7 promoter. (C) sgRNA is designed to target the genome with the standard sequence of 5′-GG(G/A)-N17/18-NGG-3′ at the 5′ of a PAM (NGG).
(PDF)

Figure S2 **Crossing strategies used in this study.** (A) Crossing Strategy 1: Injected G0 silkworms crossed with an un-injected wild-type silkworm. The main purpose of this cross was to calculate the frequency of germline transmission. (B) Crossing Strategy 2: Cross between two mosaic silkworms. The main purpose of this cross was to acquire compound heterozygotes in a one-step cross.
(PDF)

Figure S3 **Cas9/sgRNA-induced mutations at the** ***BmKMO*** **locus in** ***Bombyx mori*** **.** (A) Schematic representation of the *BmKMO* gene. Exons are shown as boxes and arrows represent the primers used to amplify the target regions. The target site location (*BmKMO*-tar) is underlined, and PAM sequences are shown in red. (B) Representative chromatograms of PCR-product sequencing in G_0_ silkworms in which indel mutations are present. (C) Sequences of indel mutations at the targeted *BmKMO* locus in G_0_ silkworms. The target sites are highlighted in green and PAM sequences are shown in red. Deletions are indicated by hyphens. The indel mutation type is noted to the right (+, insertion; -, deletion).
(PDF)

Figure S4 **Cas9/sgRNA-induced mutations at the** ***BmTH*** **locus in** ***Bombyx mori*** **.** (A) Schematic representation of the *BmTH* gene. Exons are shown as boxes and arrows represent the primers used to amplify the target regions. The target site locations (*BmTH*-tar1, *BmTH*-tar2, and *BmTH*-tar3) are underlined and PAM sequences are shown in red. (B) Representative chromatograms of PCR-product sequencing in G_0_ silkworms in which indel mutations are present. (C) Sequences of indel mutations at the targeted *BmTH* locus in G_0_ silkworms. The target sites are highlighted in green and PAM sequences are shown in red. Deletions are indicated by hyphens and insertions are shown in red lowercase letters. The indel mutation type is noted to the right (+, insertion; -, deletion).
(PDF)

Figure S5 **Cas9/sgRNA-induced mutations at the** ***Bmtan*** **locus in** ***Bombyx mori*** **.** (A) Schematic representation of the *Bmtan* gene. Exons are shown as boxes and arrows represent the primers used to amplify the target regions. The target site locations (*Bmtan*-tar1 and *Bmtan*-tar2) are underlined and PAM sequences are shown in red. (B) Sequences of indel mutations at the targeted *Bmtan* locus in G_0_ silkworms. (C) Sequences of indel mutations at the targeted *Bmtan* locus in G_0_ silkworms. The target sites are highlighted in green and PAM sequences are shown in red. Deletions are indicated by hyphens and insertions are shown in red lowercase letters. The indel mutation type is noted to the right (+, insertion; -, deletion).
(PDF)

Figure S6 **Off-targeting analysis of** ***Bm-ok*** **tar1 and tar2 sgRNA by DNA sequencing.** Typical sequencing results of the potential off-target sites are shown. Four sites each for *Bm-ok* tar1 and tar2 were tested, and no obvious multi-peaks were found. PAM sequence is in bold and potential off-target site is in lower case.
(PDF)

Table S1 **Four silkworm genes and eight associated targeting sites used in this study and oligonucleotides used to generate sgRNAs.**
(PDF)

Table S2 **List of PCR primers used for mutation characterization.**
(PDF)

Table S3 **Mutations induced by microinjection of Cas9/sgRNA in G_0_.**
(PDF)

## References

[1] Xia Q, Guo Y, Zhang Z, Li D, Xuan Z, et al (2009) Complete resequencing of 40 genomes reveals domestication events and genes in silkworm (*Bombyx*). Science 326: 433–436 10.1126/science.1176620. PubMed: 19713493 19713493 PMC3951477

[2] Yamao M, Katayama N, Nakazawa H, Yamakawa M, Hayashi Y, et al. (1999) Gene targeting in the silkworm by use of a baculovirus. Genes & Dev 13: : 511–516. PubMed: 10072379 10.1101/gad.13.5.511 PMC316505 10072379

[3] Takasu Y, Kobayashi I, Beumer K, Uchino K, Sezutsu H, et al (2010) Targeted mutagenesis in the silkworm, *Bombyx mori* using zinc finger nuclease mRNA injection. Insect Biochem Mol Biol 40: 759–765 10.1016/j.ibmb.2010.07.012. PubMed:20692340 20692340

[4] Ma S, Zhang S, Wang F, Liu Y, Liu Y, et al (2012) Highly efficient and specific genome editing in silkworm using custom TALENs. PLoS One 7: e45035.23028749 10.1371/journal.pone.0045035. PubMed: 23028749 23028749 PMC3445556

[5] Fraser MJ Jr (2012) Insect transgenesis: Current applications and future prospects. Annu Rev Entomol 57: 267–289 10.1146/annurev.ento.54.110807.090545. PubMed: 22149266 22149266

[6] Daimon T, Kiuchi T, Takasu Y (2014) Recent progress in genome engineering techniques in the silkworm, *Bombyx mori* Dev, Growth & Differ 56: : 14–25. PubMed: 24175911 10.1111/dgd.12096 24175911

[7] DiCarlo JE, Norville JE, Mali P, Rios X, Aach J, et al (2013) Genome engineering in *Saccharomyces cerevisiae* using CRISPR-Cas systems. Nucleic Acids Res 41: 4336–4343 10.1093/nar/gkt135. PubMed: 23460208 23460208 PMC3627607

[8] Tzur YB, Friedland AE, Nadarajan S, Church GM, Calarco JA, et al (2013) Heritable custom genomic modifications in *Caenorhabditis elegans* via a CRISPR-Cas9 system. Genetics 195: 1181–1185 10.1534/genetics.113.156075.PubMed: 23979579 23979579 PMC3813848

[9] Katic I, Groβhans H (2013) Targeted heritable mutation and gene conversion by Cas9-CRISPR in *Caenorhabditis elegans* . Genetics 195: 1173–1176 10.1534/genetics.113.155754. PubMed: 23979578 23979578 PMC3813846

[10] Bassett AR, Tibbit C, Ponting CP, Liu JL (2013) Highly Efficient Targeted Mutagenesis of *Drosophila* with the CRISPR/Cas9 System. Cell rep 4: 220–228 10.1016/j.celrep.2013.06.020. PubMed: 23827738 23827738 PMC3714591

[11] Gratz SJ, Cummings AM, Nguyen JN, Hamm DC, Donohue LK, et al (2013) Genome engineering of *Drosophila* with the CRISPR RNA-guided Cas9 nuclease. Genetics 194: 1029–1035 10.1534/genetics.113.152710. PubMed: 23709638 23709638 PMC3730909

[12] Yu Z, Ren M, Wang Z, Zhang B, Rong YS, et al (2013) Highly efficient genome modifications mediated by CRISPR/Cas9 in *Drosophila* . Genetics 195: 289–291 10.1534/genetics.113.153825. PubMed: 23833182 23833182 PMC3761309

[13] Hwang WY, Fu Y, Reyon D, Maeder ML, Tsai SQ, et al (2013) Efficient genome editing in zebrafish using a CRISPR-Cas system. Nat Biotechnol 31: 227–229 10.1038/nbt.2501. PubMed: 23360964 23360964 PMC3686313

[14] Cho SW, Kim S, Kim JM, Kim JS (2013) Targeted genome engineering in human cells with the Cas9 RNA-guided endonuclease. Nat Biotechnol 31: 230–232 10.1038/nbt.2507. PubMed: 23360966 23360966

[15] Cong L, Ran FA, Cox D, Lin S, Barretto R, et al (2013) Multiplex genome engineering using CRISPR/Cas systems. Science 339: 819–823 10.1126/science.1231143. PubMed: 23287718 23287718 PMC3795411

[16] Mali P, Yang L, Esvelt KM, Aach J, Guell M, et al (2013) RNA-guided human genome engineering via Cas9. Science 339: 823–826 10.1126/science.1232033. PubMed: 23287722 23287722 PMC3712628

[17] Wang H, Yang H, Shivalila CS, Dawlaty MM, Cheng AW, et al (2013) One-step generation of mice carrying mutations in multiple genes by CRISPR/Cas-mediated genome engineering. Cell 153: 910–918 10.1016/j.cell.2013.04.025. PubMed: 23643243 23643243 PMC3969854

[18] Li JF, Norville JE, Aach J, McCormack M, Zhang D, et al (2013) Multiplex and homologous recombination-mediated genome editing in *Arabidopsis* and *Nicotiana benthamiana* using guide RNA and Cas9. Nat Biotechnol 31: 688–691 10.1038/nbt.2654. PubMed: 23929339 23929339 PMC4078740

[19] Wang Y, Li Z, Xu J, Zeng B, Ling L, et al. (2013) The CRISPR/Cas System mediates efficient genome engineering in *Bombyx mori*. Cell Res doi: 10.1038/cr.2013.146. 341 PubMed: 24165890 10.1038/cr.2013.146 PMC3847576 24165890

[20] Wiedenheft B, Sternberg SH, Doudna JA (2012) RNA-guided genetic silencing systems in bacteria and archaea. Nature 482: 331–338 10.1038/nature10886. PubMed: 22337052 22337052

[21] Buckner JS, Newman SM (1990) Uric acid storage in the epidermal cells of *Manduca sexta*: Localization and movement during the larval-pupal transformation. J Insect Physiol 36: 219–229.

[22] Wang L, Kiuchi T, Fujii T, Daimon T, Li M, et al (2013) Mutation of a novel ABC transporter gene is responsible for the failure to incorporate uric acid in the epidermis of ok mutants of the silkworm, *Bombyx mori* . Insect Biochem Mol Biol 43: 562–571 10.1016/j.ibmb.2013.03.011. PubMed: 23567590 23567590

[23] Kikkawa H (1953) Biochemical Genetics of *Bombyx mori* (Silkworm). Adv Genet 5: : 107–140. PubMed: 13040134 10.1016/s0065-2660(08)60407-1 13040134

[24] Seitz V, Clermont A, Wedde M, Hummel M, Vilcinskas A, et al. (2003) Identification of immunorelevant genes from greater wax moth (*Galleria mellonella*) by a subtractive hybridization approach. Dev Comp Immunol 27: : 207–215. PubMed: 12590972 10.1016/s0145-305x(02)00097-6 12590972

[25] De Gregorio E, Spellman PT, Rubin GM, Lemaitre B (2001) Genome-wide analysis of the *Drosophila* immune response by using oligonucleotide microarrays. Proc Natl Acad Sci 98: : 12590–12595. PubMed: 11606746 10.1073/pnas.221458698 PMC60098 11606746

[26] True JR (2003) Insect melanism: the molecules matter. Trends Ecol Evol 18: 640–647.

[27] Wittkopp PJ, Carroll SB, Kopp A (2003) Evolution in black and white: genetic control of pigment patterns in *Drosophila* Trends Genet 19: : 495–504. PubMed: 12957543 10.1016/S0168-9525(03)00194-X 12957543

[28] Zhao AC, Long DP, Ma SY, Xu LX, Zhang MR, et al (2012) Efficient strategies for changing the diapause character of silkworm eggs and for the germline transformation of diapause silkworm strains. Insect Science 19(2): 172–182.

[29] Chang N, Sun C, Gao L, Zhu D, Xu X, et al (2013) Genome editing with RNA-guided Cas9 nuclease in Zebrafish embryos. Cell Res 23: 465–472 10.1038/cr.2013.45. PubMed: 23528705 23528705 PMC3616424

[30] Tamura T, Kuwabara N, Uchino K, Kobayashi I, Kanda T (2007) An improved DNA injection method for silkworm eggs drastically increases the efficiency of producing transgenic silkworms. Journal of Insect Biotechnol Sericol 76: 155–159.

[31] Xiao A, Cheng Z, Kong L, Zhu Z, Lin S, et al. (2014) CasOT: a genome-wide Cas9/sgRNA off-target searching tool. Bioinformatics, Online publish (http://bioinformatics.oxfordjournals.org/content/early/2014/01/02/bioinformatics.btt764.short)PubMed: 24389662. 10.1093/bioinformatics/btt764 24389662

[32] Fu Y, Foden JA, Khayter C, Maeder ML, Reyon D, et al (2013) High-frequency off-target mutagenesis induced by CRISPR-Cas nucleases in human cells, Nat Biotechnol. 31: 822–826 10.1038/nbt.2623. PubMed:23792628 PMC3773023 23792628

