# Supplementary material for: Heritable Genome Editing with CRISPR/Cas9 in the Silkworm, Bombyx mori
Source: PLoS One. 2014 Jul 11;9(7):e101210. doi: 10.1371/journal.pone.0101210 (PMC4094479; doi:10.1371/journal.pone.0101210)
Supplement: Figure S1 — Schematic illustration of the CRISPR/Cas9 system and the genome engineering process. (A) Map of the pSP6-2sNLS-SpCas9 vector. (B) Map of the pMD19-T sgRNA scaffold vector used to produce sgRNA. The transcription of Cas9 flanked by two nuclear localization sequences (NLS) is driven by the SP6 promoter. sgRNA transcription is driven by the T7 promoter. (C) sgRNA is designed to target the genome with the standard sequence of 5′-GG(G/A)-N17/18-NGG-3′ at the 5′ of a PAM (NGG). (PDF) [file pone.0101210.s001.pdf]

Figure S1

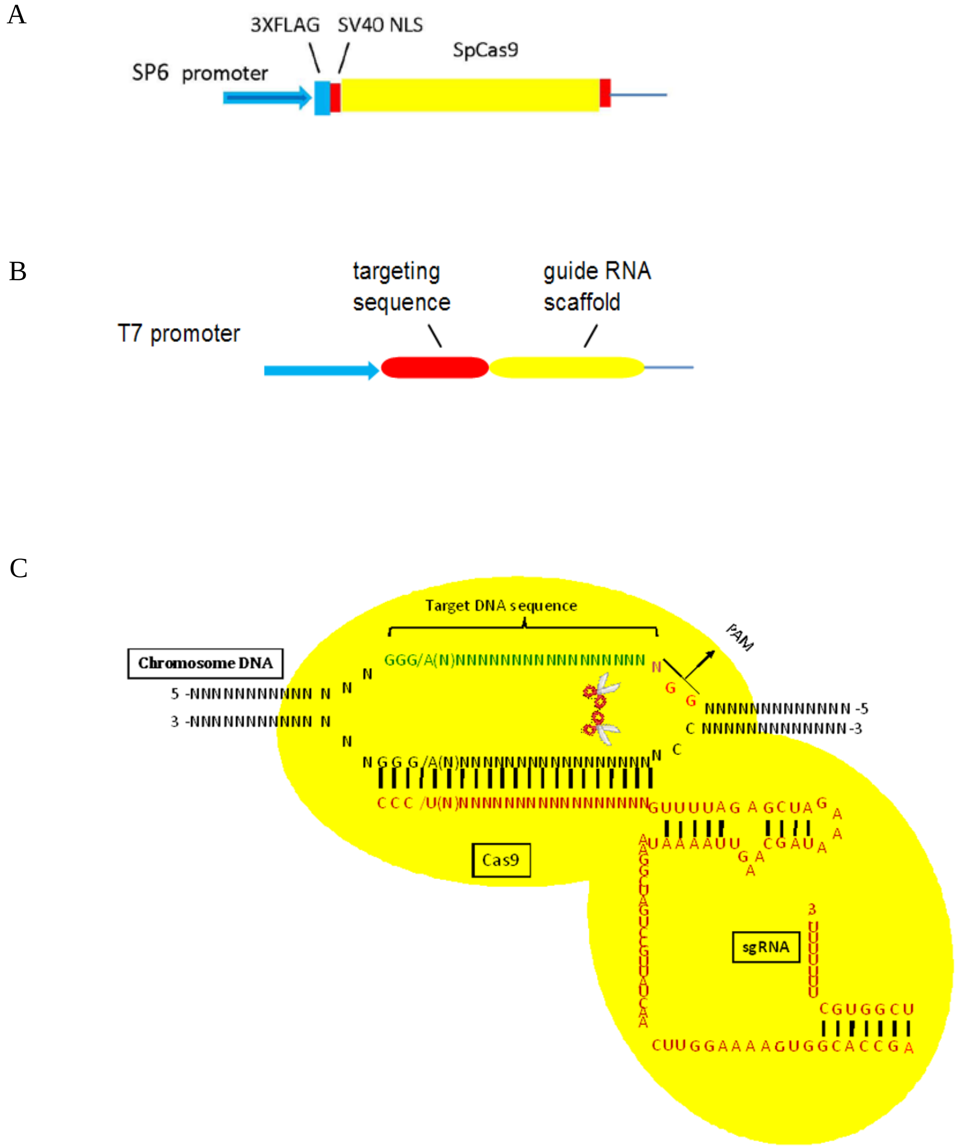

Figure S1 Schematic illustration of the CRISPR/Cas9 system and the genetic engineering process. (A) Map of the pSP6-2sNLS-SpCas9 vector. (B) Map of the pMD19-T sgRNA scaffold vector used to produce sgRNA. The transcription of Cas9 flanked by two nuclear localization sequences (NLS) is driven by the SP6 promoter. sgRNA transcription is driven by the T7 promoter. (C) sgRNA is designed to target the genome with the standard sequence of 5’-GG(G/A)-N17/18-NGG-3’ at the 5’ of a PAM (NGG).
