# Supplementary material for: Heritable Genome Editing with CRISPR/Cas9 in the Silkworm, Bombyx mori
Source: PLoS One. 2014 Jul 11;9(7):e101210. doi: 10.1371/journal.pone.0101210 (PMC4094479; doi:10.1371/journal.pone.0101210)
Supplement: Figure S2 — Crossing strategies used in this study. (A) Crossing Strategy 1: Injected G0 silkworms crossed with an un-injected wild-type silkworm. The main purpose of this cross was to calculate the frequency of germline transmission. (B) Crossing Strategy 2: Cross between two mosaic silkworms. The main purpose of this cross was to acquire compound heterozygotes in a one-step cross. (PDF) [file pone.0101210.s002.pdf]

Figure S2

A

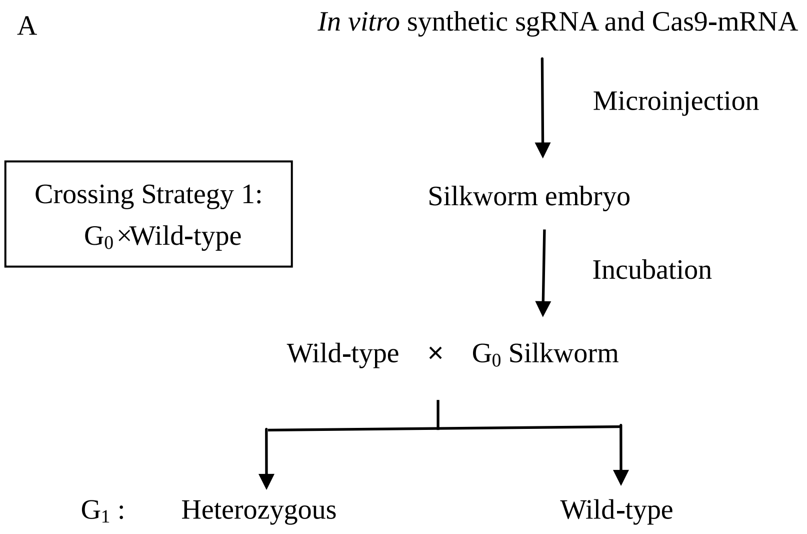

B

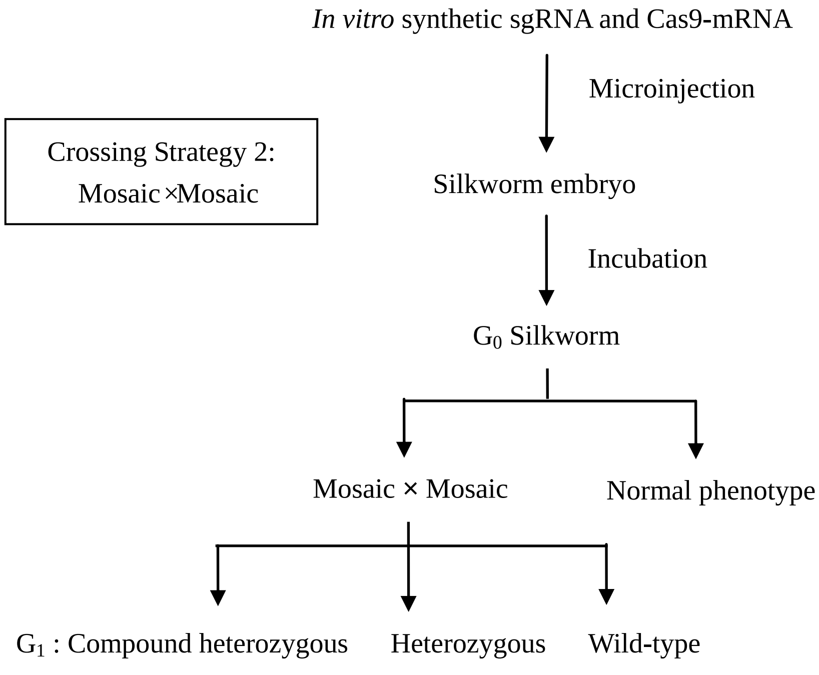

Figure S2 Crossing strategies used in this study. (A) Crossing Strategy 1: Injected G_0 silkworms crossed with an un-injected wild-type silkworm. The main purpose of this cross was to calculate the frequency of germline transmission. (B) Crossing Strategy 2: Cross between two mosaic silkworms. The main purpose of this cross was to acquire compound heterozygotes in a one-step cross.
