# Supplementary material for: Heritable Genome Editing with CRISPR/Cas9 in the Silkworm, Bombyx mori
Source: PLoS One. 2014 Jul 11;9(7):e101210. doi: 10.1371/journal.pone.0101210 (PMC4094479; doi:10.1371/journal.pone.0101210)
Supplement: Figure S3 — Cas9/sgRNA-induced mutations at the BmKMO locus in Bombyx mori . (A) Schematic representation of the BmKMO gene. Exons are shown as boxes and arrows represent the primers used to amplify the target regions. The target site location (BmKMO-tar) is underlined, and PAM sequences are shown in red. (B) Representative chromatograms of PCR-product sequencing in G0 silkworms in which indel mutations are present. (C) Sequences of indel mutations at the targeted BmKMO locus in G0 silkworms. The target sites are highlighted in green and PAM sequences are shown in red. Deletions are indicated by hyphens. The indel mutation type is noted to the right (+, insertion; -, deletion). (PDF) [file pone.0101210.s003.pdf]

Figure S3

A

B

C

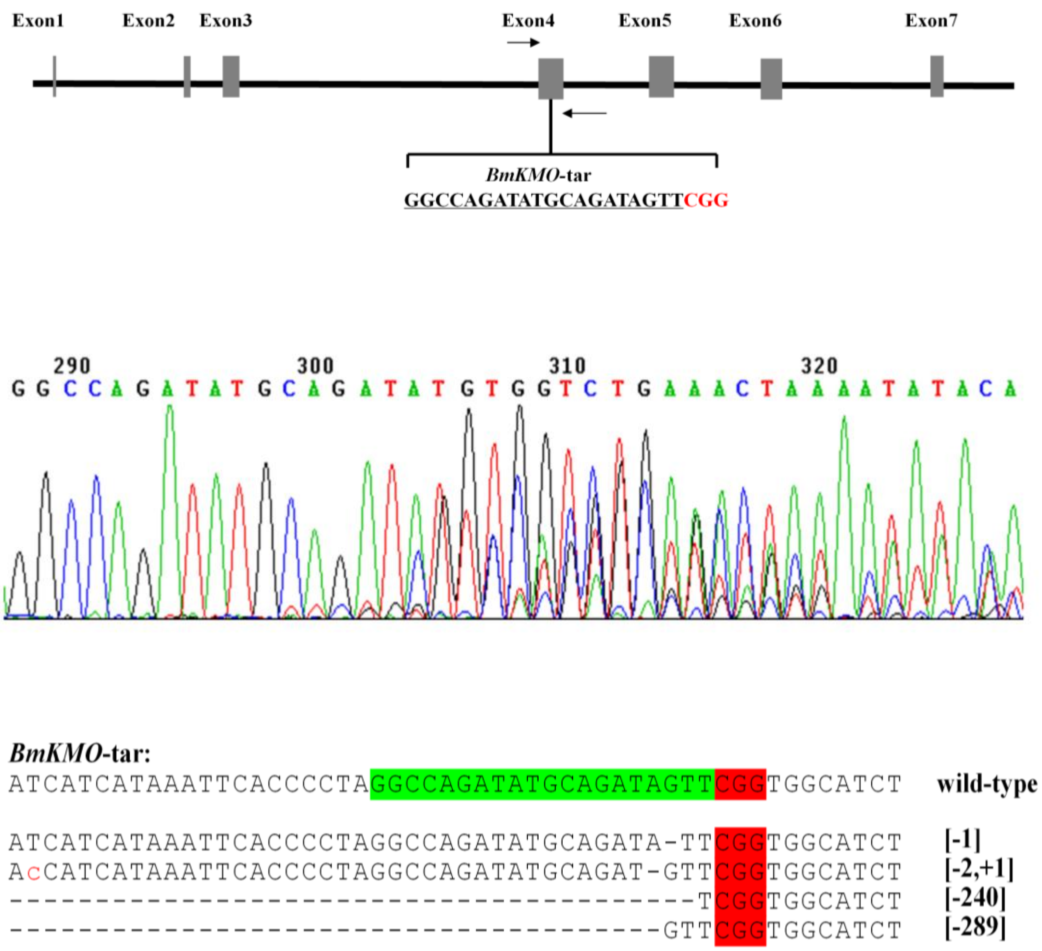

Figure S3 Cas9/sgRNA-induced mutations at the *BmKMO* locus in *Bombyx mori*. (A) Schematic representation of the *BmKMO* gene. Exons are shown as boxes and arrows represent the primers used to amplify the target regions. The target site location (*BmKMO*-tar) is underlined, and PAM sequences are shown in red. (B) Representative chromatograms of PCR-product sequencing in G_0 silkworms in which indel mutations are present. (C) Sequences of indel mutations at the targeted *BmKMO* locus in G_0 silkworms. The target sites are highlighted in green and PAM sequences are shown in red. Deletions are indicated by hyphens. The indel mutation type is noted to the right (+, insertion; -, deletion).
