# Supplementary material for: Heritable Genome Editing with CRISPR/Cas9 in the Silkworm, Bombyx mori
Source: PLoS One. 2014 Jul 11;9(7):e101210. doi: 10.1371/journal.pone.0101210 (PMC4094479; doi:10.1371/journal.pone.0101210)
Supplement: Figure S4 — Cas9/sgRNA-induced mutations at the BmTH locus in Bombyx mori . (A) Schematic representation of the BmTH gene. Exons are shown as boxes and arrows represent the primers used to amplify the target regions. The target site locations (BmTH-tar1, BmTH-tar2, and BmTH-tar3) are underlined and PAM sequences are shown in red. (B) Representative chromatograms of PCR-product sequencing in G0 silkworms in which indel mutations are present. (C) Sequences of indel mutations at the targeted BmTH locus in G0 silkworms. The target sites are highlighted in green and PAM sequences are shown in red. Deletions are indicated by hyphens and insertions are shown in red lowercase letters. The indel mutation type is noted to the right (+, insertion; -, deletion). (PDF) [file pone.0101210.s004.pdf]

Figure S4

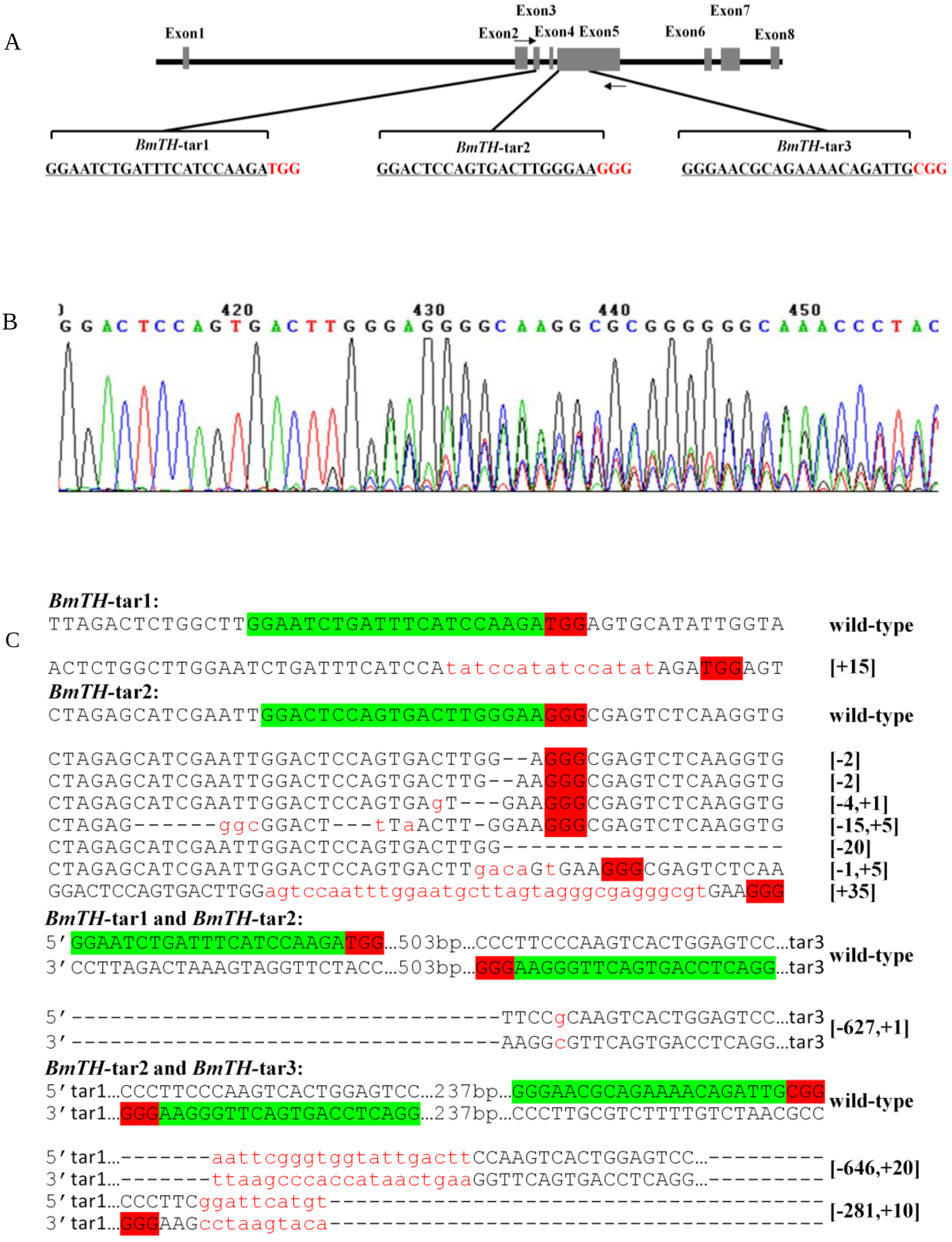

Figure S4 Cas9/sgRNA-induced mutations at the *BmTH* locus in *Bombyx mori*. (A)

Schematic representation of the *BmTH* gene. Exons are shown as boxes and arrows represent the primers used to amplify the target regions. The target site locations (*BmTH*-tar1, *BmTH*-tar2, and *BmTH*-tar3) are underlined and PAM sequences are shown in red. (B) Representative chromatograms of PCR-product sequencing in G_0 silkworms in which indel mutations are present. (C) Sequences of indel mutations at the targeted *BmTH* locus in G_0 silkworms. The target sites are highlighted in green and PAM sequences are shown in red. Deletions are indicated by hyphens and insertions are shown in red lowercase letters. The indel mutation type is noted to the right (+, insertion; -, deletion).
