# Supplementary material for: Heritable Genome Editing with CRISPR/Cas9 in the Silkworm, Bombyx mori
Source: PLoS One. 2014 Jul 11;9(7):e101210. doi: 10.1371/journal.pone.0101210 (PMC4094479; doi:10.1371/journal.pone.0101210)
Supplement: Figure S5 — Cas9/sgRNA-induced mutations at the Bmtan locus in Bombyx mori . (A) Schematic representation of the Bmtan gene. Exons are shown as boxes and arrows represent the primers used to amplify the target regions. The target site locations (Bmtan-tar1 and Bmtan-tar2) are underlined and PAM sequences are shown in red. (B) Sequences of indel mutations at the targeted Bmtan locus in G0 silkworms. (C) Sequences of indel mutations at the targeted Bmtan locus in G0 silkworms. The target sites are highlighted in green and PAM sequences are shown in red. Deletions are indicated by hyphens and insertions are shown in red lowercase letters. The indel mutation type is noted to the right (+, insertion; -, deletion). (PDF) [file pone.0101210.s005.pdf]

Figure S5

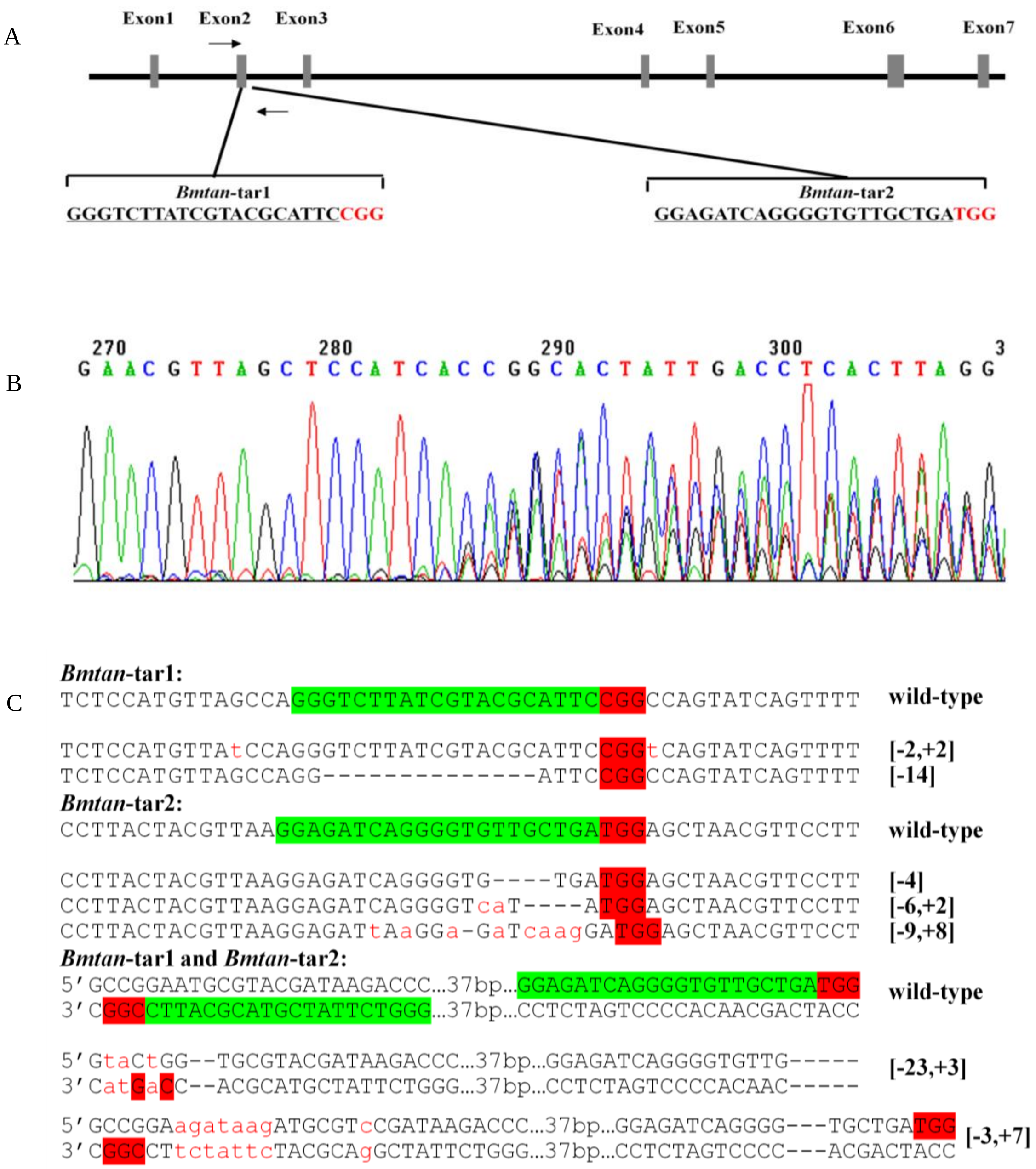

Figure S5 Cas9/sgRNA-induced mutations at the *Bmtan* locus in *Bombyx mori*. (A) Schematic representation of the *Bmtan* gene. Exons are shown as boxes and arrows represent the primers used to amplify the target regions. The target site locations (*Bmtan*-tar1 and *Bmtan*-tar2) are underlined and PAM sequences are shown in red. (B) Sequences of indel

mutations at the targeted *Bmtan* locus in G_0 silkworms. (C) Sequences of indel mutations at the targeted *Bmtan* locus in G_0 silkworms. The target sites are highlighted in green and PAM sequences are shown in red. Deletions are indicated by hyphens and insertions are shown in red lowercase letters. The indel mutation type is noted to the right (+, insertion; -, deletion).
