# Supplementary material for: Heritable Genome Editing with CRISPR/Cas9 in the Silkworm, Bombyx mori
Source: PLoS One. 2014 Jul 11;9(7):e101210. doi: 10.1371/journal.pone.0101210 (PMC4094479; doi:10.1371/journal.pone.0101210)
Supplement: Figure S6 — Off-targeting analysis of Bm-ok tar1 and tar2 sgRNA by DNA sequencing. Typical sequencing results of the potential off-target sites are shown. Four sites each for Bm-ok tar1 and tar2 were tested, and no obvious multi-peaks were found. PAM sequence is in bold and potential off-target site is in lower case. (PDF) [file pone.0101210.s006.pdf]

Figure S6

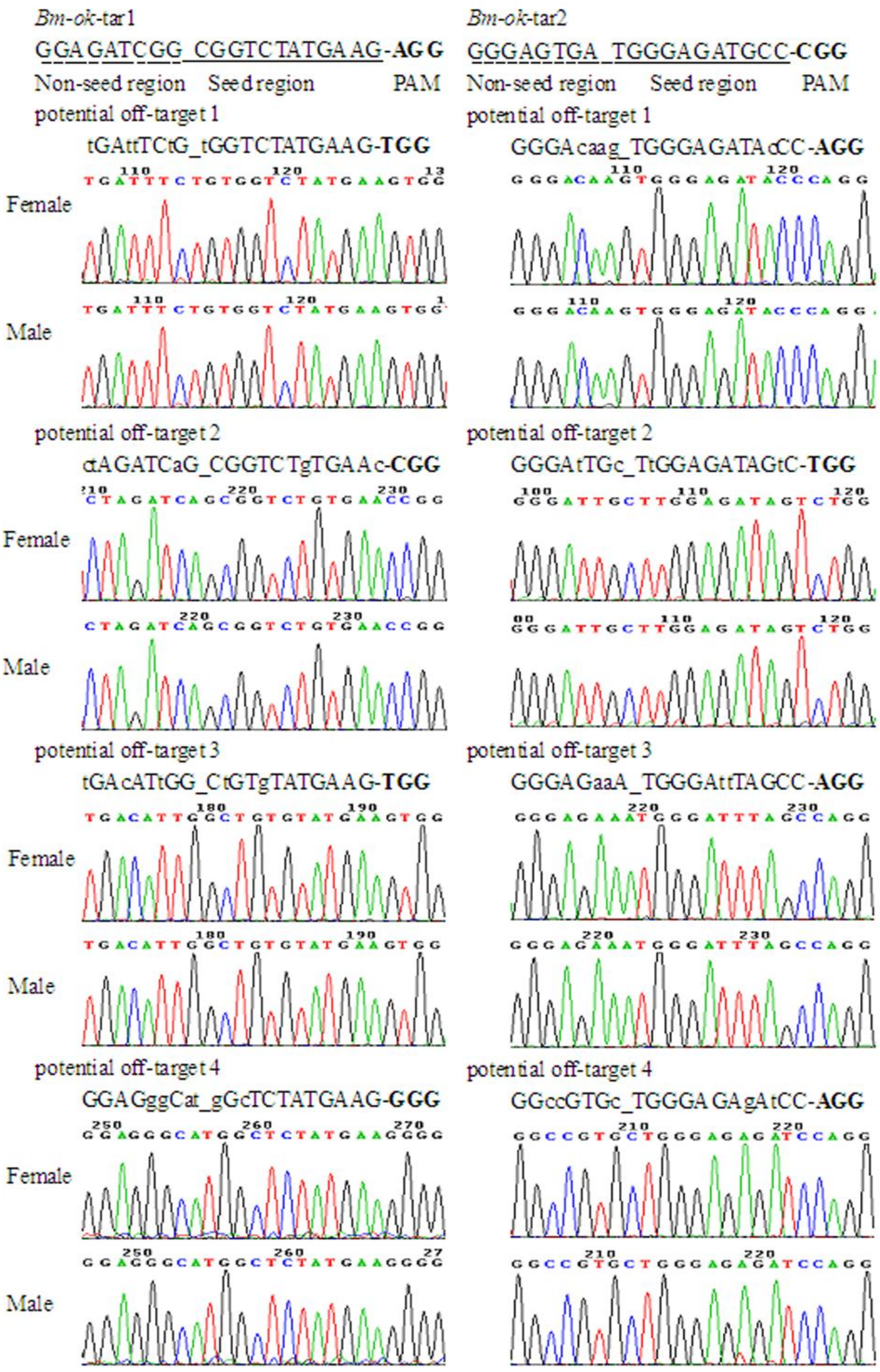

Figure S6 Off-target analysis of *Bm-ok* tar1 and tar2 sgRNA by DNA sequencing. Typical sequencing results of the potential off-target sites are shown. Four sites each for *Bm-ok* tar1 and tar2 were tested, and no obvious multi-peaks were found. PAM sequence is in bold and potential off-target site is in lower case.
