# Supplementary material for: Heritable Genome Editing with CRISPR/Cas9 in the Silkworm, Bombyx mori
Source: PLoS One. 2014 Jul 11;9(7):e101210. doi: 10.1371/journal.pone.0101210 (PMC4094479; doi:10.1371/journal.pone.0101210)
Supplement: Table S1 — Four silkworm genes and eight associated targeting sites used in this study and oligonucleotides used to generate sgRNAs. (PDF) [file pone.0101210.s007.pdf]

**Table S1** Four silkworm genes and eight associated targeting sites used in this study and oligonucleotides used to generate sgRNAs

| Target gene | Target site (5'–3') (PAM is underlined) | | Oligonucleotide 1 (5'–3') | Oligonucleotide 2 (5'–3') |
|---|---|---|---|---|
| *Bm-ok* | tar1 | GGAGATCGGCGGTCTAT GAAGAGG | TAATACGACTCACTATAGGAGAT CGGCGGTCTATGAAGGTTTTAG AGCTAGAAATAGC | AAAAAAAGC ACCGACTCG GTGCCAC |
| | tar2 | GGGAGTGATGGGAGAT AGCCCGG | TAATACGACTCACTATAGGGAG TGATGGGAGATAGCCGTTTTAG AGCTAGAAATAGC | Same as above |
| *BmKMO* | tar | GGCCAGATATGCAGATA GTTCGG | TAATACGACTCACTATAGGCCA GATATGCAGATAGTTGTTTTAGA GCTAGAAATAGC | Same as above |
| *BmTH* | tar1 | GGAATCTGATTTCATCC AAGATGG | TAATACGACTCACTATAGGAATC TGATTTCATCCAAGAGTTTTAG AGCTAGAAATAGC | Same as above |
| | tar2 | GGACTCCAGTGACTTG GGAAGGG | TAATACGACTCACTATAGGACTC CAGTGACTTGGGAAGTTTTAGA GCTAGAAATAGC | Same as above |
| | tar3 | GGGAACGCAGAAAACA GATTGCGG | TAATACGACTCACTATAGGGAA CGCAGAAAACAGATTGGTTTTA GAGCTAGAAATAGC | Same as above |
| *Bmtan* | tar1 | GGGTCTTATCGTACGCA TTCCGG | TAATACGACTCACTATAGGGTCT TATCGTACGCATTCGTTTTAGAG CTAGAAATAGC | Same as above |
| | tar2 | GGAGATCAGGGGTGTT GCTGATGG | TAATACGACTCACTATAGGAGAT CAGGGGTGTTGCTGAGTTTTAG AGCTAGAAATAGC | Same as above |
