# Supplementary material for: Heritable Genome Editing with CRISPR/Cas9 in the Silkworm, Bombyx mori
Source: PLoS One. 2014 Jul 11;9(7):e101210. doi: 10.1371/journal.pone.0101210 (PMC4094479; doi:10.1371/journal.pone.0101210)
Supplement: Table S2 — List of PCR primers used for mutation characterization. (PDF) [file pone.0101210.s008.pdf]

Table S2 List of PCR primers used for mutation characterization

Target gene	Primer name	Primer sequence (5'–3') Forward and Reverse
Bm-ok	*Bmok*-F	GACGAGAACGATGACCGGAA
	Bmok-R	GAATACAATCTTAAGCTACCCACGC
BmKMO	*BmKMO*-F	ATCATTGCGCAAACACTGGT
	BmKMO-R	AGGTGGAAATTGCCAAACTTTTT
BmTH	*BmTH*-F	TGACAAATGGATGTTTTCCGTGAA
	BmTH-R	GGTACGAATATGTCTGCTGCCTGT
Bmtan	*Bmtan*-F	CGCACATTCTCCTCCATCATCAA
	Bm*tan*-R	ACTTTAGCAACTGCGTGCCTTCT
