# Supplementary material for: Heritable Genome Editing with CRISPR/Cas9 in the Silkworm, Bombyx mori
Source: PLoS One. 2014 Jul 11;9(7):e101210. doi: 10.1371/journal.pone.0101210 (PMC4094479; doi:10.1371/journal.pone.0101210)
Supplement: Table S3 — Mutations induced by microinjection of Cas9/sgRNA in G0. (PDF) [file pone.0101210.s009.pdf]

Table S3 Mutations induced by microinjection of Cas9/sgRNA in G_0

Target gene	Embryos Injected (n)	Total sequencing number (n)	Mutant number by sequencing (n)	Frequency of mutation in G_0
BmKMO	437	20	7	35.0% (7/20)
BmTH	136	24	8	33.3% (8/24)
Bmtan	473	6	1	16.7% (1/6)
